# A novel signature of two long non-coding RNAs in BRCA mutant ovarian cancer to predict prognosis and efficiency of chemotherapy

**DOI:** 10.1186/s13048-020-00712-w

**Published:** 2020-09-19

**Authors:** Yinglian Pan, Li Ping Jia, Yuzhu Liu, Yiyu Han, Qian Li, Qin Zou, Zhongpei Zhang, Jin Huang, Qingchun Deng

**Affiliations:** 1 Department of Medical Oncology, The First Affiliated Hospital of Hainan Medical University, Haikou, 570102 Hainan China; 2 Department of Gynecology, The Second Affiliated Hospital of Hainan Medical University, Haikou, China; 3 Department of Clinical Laboratory, Wuhan Fourth Hospital, Puai Hospital, Tongji Medical College, Huazhong University of Science and Technology Wuhan, Wuhan, China

**Keywords:** Ovarian cancer, Long non-coding RNA, Prognostic biomarker, Mutations, *BRCA1/2* gene, Chemotherapy, Efficiency

## Abstract

**Background:**

In this study we aimed to identify a prognostic signature in *BRCA1/2* mutations to predict disease progression and the efficiency of chemotherapy ovarian cancer (OV), the second most common cause of death from gynecologic cancer in women worldwide.

**Methods:**

Univariate Cox proportional-hazards and multivariate Cox regression analyses were used to identifying prognostic factors from data obtained from The Cancer Genome Atlas (TCGA) database. The area under the curve of the receiver operating characteristic curve was assessed, and the sensitivity and specificity of the prediction model were determined.

**Results:**

A signature consisting of two long noncoding RNAs(lncRNAs), Z98885.2 and AC011601.1, was selected as the basis for classifying patients into high and low-risk groups (median survival: 7.2 years vs. 2.3 years). The three-year overall survival (OS) rates for the high- and low-risk group were approximately 38 and 100%, respectively. Chemotherapy treatment survival rates indicated that the high-risk group had significantly lower OS rates with adjuvant chemotherapy than the low-risk group. The one-, three-, and five-year OS were 100, 40, and 15% respectively in the high-risk group. The survival rate of the high-risk group declined rapidly after 2 years of OV chemotherapy treatment. Multivariate Cox regression associated with other traditional clinical factors showed that the 2-lncRNA model could be used as an independent OV prognostic factor. Analyses of data from the Kyoto Encyclopedia of Genes and Genomes (KEGG) and Gene Ontology (GO) indicated that these signatures are pivotal to cancer development.

**Conclusion:**

In conclusion, Z98885.2 and AC011601.1 comprise a novel prognostic signature for OV patients with *BRCA1/2* mutations, and can be used to predict prognosis and the efficiency of chemotherapy.

## Introduction

By 2020, more than 300,000 new cases of OV are expected to occur worldwide, with more than 190,000 deaths expected [1]. Early symptoms are difficult to interpret correctly, and peritoneal metastasis often occurs before symptoms appear. Reports indicate that 60–70% of patients are diagnosed at advanced stages. The OV mortality rate has always been the highest of the female reproductive tract malignancies [2]. Therefore, early diagnosis and treatment are crucial to improving the quality of life and survival rate of OV patients.

Tumor cell abatement and platinum-based chemotherapy after surgery are the standard methods of treatment. The breast cancer susceptibility genes, *BRCA1*/*2*, are critical tumor suppressor genes [3]. Patients with germline or system *BRCA 1/2* mutations in homologous recombination genes have a better prognosis, including higher sensitivity to platinum, longer disease-free survival, and longer survival [4]. Cancers cells with *BRCA1/2* mutations are extremely sensitive to chemotherapy drugs such as platinum, that induce DNA double-strand breaks [5]. *BRCA1/2* mutation status is an important prognostic factor in OV patients. OV patients with *BRCA1/2* mutations have a better prognosis than those with wild-type *BRCA1/2* genes, both in terms of progression-free survival and total survival, and mutations in *BRCA2* may have a better prognosis than *BRCA1* mutations [6]. PARP inhibitors are effective in OV patients with *BRCA 1/2* mutations or other homologous recombination defects. *BRCA1/2* is therefore a vital biomarker for the evaluation of the risk of OV and other related cancers, and also serves as a biomarker for personalizing treatment [7, 8].

Long noncoding RNAs (lncRNAs) are a family of nonprotein-coding RNAs of 200–100,000 nucleotides [9]. Recent studies have demonstrated that abnormal expression of various lncRNAs has been detected in large clinical biopsy specimens. The presence of lncRNAs is closely related to the recurrence, metastasis and prognosis of various tumors, suggesting that lncRNAs can be used as new potential molecular markers for tumor prognosis. Perez et al. [10] reported that lncRNA expression differs between OV and healthy tissues; however, the functional differences involved were not identified. A separate study into 115 lncRNAs showed that in OV SKOV3 cells, estrogen could induce the production of lncRNAs, regulating cell migration and invasion during estrogen signaling. These studies indicate that lncRNAs plays a vital role in the development of OV [11].

Emerging evidence suggests that lncRNAs have the prognostic potential to act as multidimensional transcriptome signature. The aim of this study was to identify a novel lncRNA prognostic biomarker to provide potentially new and accurate biological indicators for the early diagnosis and monitoring of prognosis of ovarian cancer bearing *BRCA1/2* mutations.

## Materials and methods

### Clinical cohorts and different types of molecular data

**Fig. 1:**
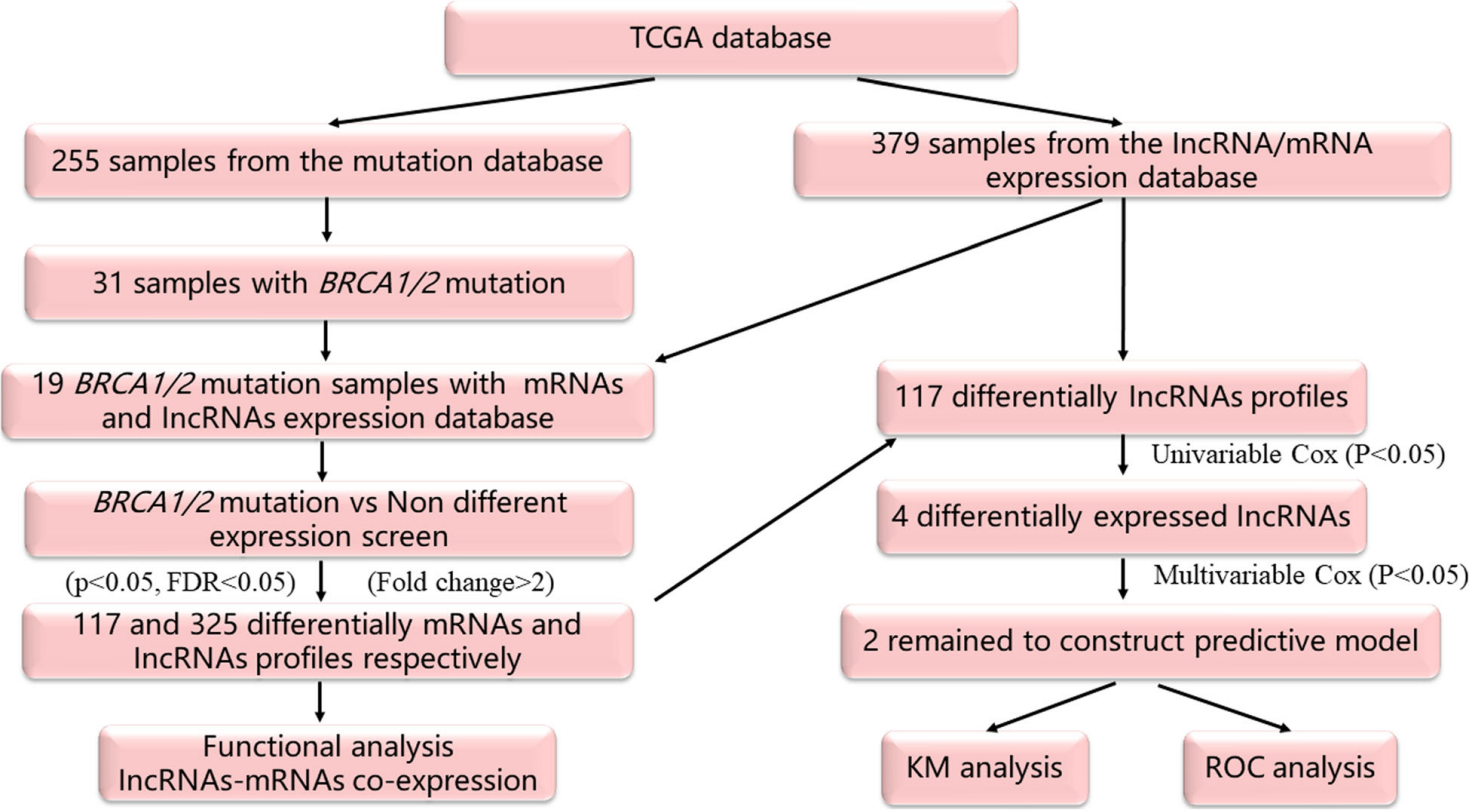
Study protocol. The order of analyses to develop the risk score model and validate the efficiency of the signature to predict prognostic outcomes

The Cancer Genome Atlas [12] (TCGA; https://cancergenome.nih.gov/) was used to download clinical information and different types of molecular data, including 255 samples in a somatic mutation dataset and 379 samples in an mRNA and lncRNA expression dataset; clinical information was available for 375 patients. The technical route for selecting lncRNA signal signatures to predict prognostic outcomes is depicted in Fig. 1.

### Description of *BRCA1*/ *2* mutated dataset

The somatic mutation data in var. scan format was downloaded. All genes harboring nonsynonymous or nonsense mutations were derived from within among these datasets. Infrequently mutated genes were excluded on the base of a 5% mutation frequency threshold, and data regarding mutated genes were curated into a binary matrix from which 20 patients with *BRCA1/2* mutations were identified. GenVisR (http://bioconductor.org/packages/release/bioc/html/GenVisR.html) was used to the visualize mutations graphically as a waterfall image.

### Identification of the differentially expressed mRNAs and lncRNAs

Differentially expressed mRNAs and lncRNAs were identified using the edgeR software to analyze 31 patients with *BRCA1/2* mutations and 224 patients without *BRCA1*/*2* mutations. Fold changes (log_2_ absolute) ≥2, *P* < 0.05, FDR < 0.05 were taken to indicate statistical significance.

### Construction of an lncRNA signature from the *BRCA1*/*2* mutated dataset

The signature module was constructed as previously described [13–17]. The Univariate Cox regression analysis was used to assess the combination of survival time, and the constant expression degree of each lncRNA in the *BRCA1*/*2* mutated data set. To filter out the most useful predictive prognostic lncRNAs, multivariate Cox regression analysis was subsequently performed to establish a model to evaluate the prognosis in accordance with the following equation:

$$ \mathrm{Risk}\ \mathrm{Score}\left(\mathrm{RS}\right)=\sum \limits_{i=1}^N{Ex}_i\ast {Coef}_i $$

Where, *N* is the representative number of lncRNAs in prognosis, *Ex*_*i*_ is the definition value of the lncRNAs, and *Coef*_*i*_ is a single factor of Cox regression coefficient. *Risk Score (RS)* is the multi-node weighted sum of risk scores.

### Statistical analysis

LncRNAs were selected to establish the risk model, and the individuals with *BRCA1*/*2* mutation were divided into high- and low-risk groups using the median risk score and cut-off values. The effective prognostic potency and effects of chemotherapy treatment as identified using the lncRNA signature were investigated using Kaplan-Meier (KM) survival analysis and receiver operating characteristic (ROC) analysis. Multivariable Cox regression was performed to validate the performance of the signature for the prediction of survival. The RNAs package was used in the R program to create a nomogram, including grading and age, as these variables are typically included in the prognostic models for most *BRCA1*/*2* mutant groups. Nomograms were constructed on the basis of coefficients of the multivariate Cox regression model. All assessments were carried out using R software (https://cloud.r-project.org/)(version 3.5.1) with pROC and survival packages downloaded from Bioconductor (https://bioconductor.org). For all analysis *P* < 0.05 was considered significant.

### Functional analysis of differentially expressed mRNAs

Gene Ontology (GO) analysis, comprising biological processes, molecular functions, and cellular component, was performed from Kyoto Encyclopedia of Genes and Genomes (KEGG). The functions associated with the signatures of the differentially expressed genes were predicted using the DAVID Bioinformatics Tool (https://david.ncifcrf.gov/, version 6.8).

## Results

### Patient characteristics

A total of 375 patients were clinically and pathologically diagnosed with OV. In accordance with the International Federation of Gynecology and Obstetrics (FIGO) classification catalogue [18–20], the grading of endometrioid carcinomas was identical to that of uterine endometrioid carcinomas and was of prognostic and therapeutic significance. In total, 6, 68, and 301 patients were diagnosed with at grades 1, 2, and 3, respectively. Clinical data for all patients is shown in Table 1. The flowchart for the analysis of the selected lncRNA and mRNA signatures is shown in Fig. 1.

**Table 1:** Summary of patient demographics and characteristics

Characteristic	Sample
**Gender**	
male	0
female	375
**Age**	
Median	60
Range	30 ~ 87
**Grade**	
G1	6
G2	68
G3	301
**Vital status**	
Living	145
Dead	230
***BRCA1***	13
***BRCA2***	6

### Differentially expressed mRNAs and lncRNAs

**Fig. 2:**
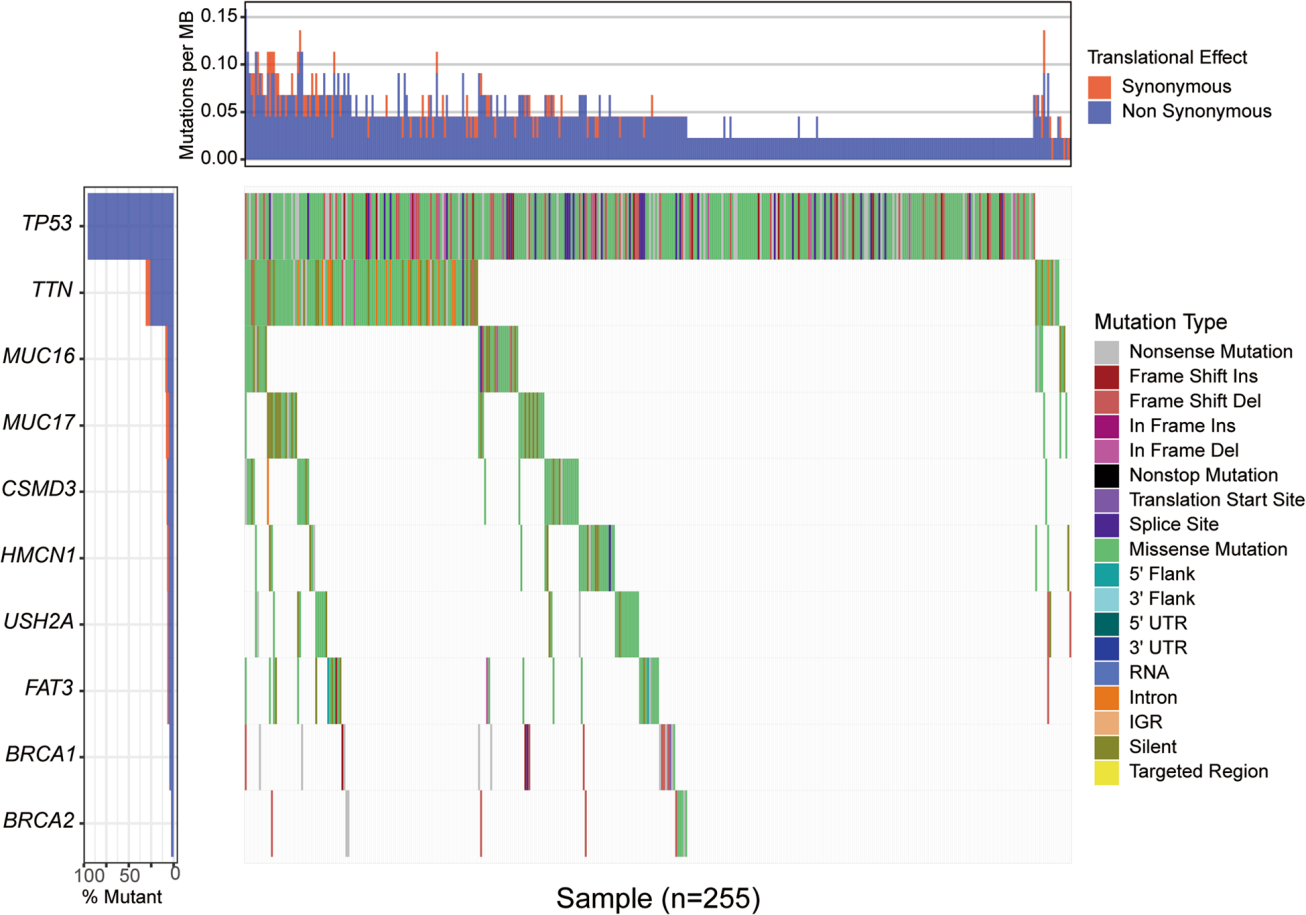
The waterfall image of partial mutations in OV. GenVisR was used to visualize mutations graphically, using the waterfall image

A total of 20 patients with *BRCA1* mutations and 11 patients with *BRCA2* mutations were identified from 255 samples in the somatic mutation data. A total of 19,495 mRNAs and 14,589 lncRNAs were identified from the 31 patients with *BRCA1*/*2* mutations (Table S1). Using fold |changes| ≥2 and *P* < 0.05 as cutoffs, we identified 325 differentially expressed mRNAs (149 downregulated and 176 upregulated) and 117 differentially expressed lncRNAs (24 downregulated and 93 upregulated), as shown in the heatmap (Fig. 2). The distribution of differentially expressed mRNAs and lncRNAs is shown in the volcano plot map (Figure S1).

### Construction of the prognostic *BRCA1*/*2* lncRNA signature

**Fig. 3:**
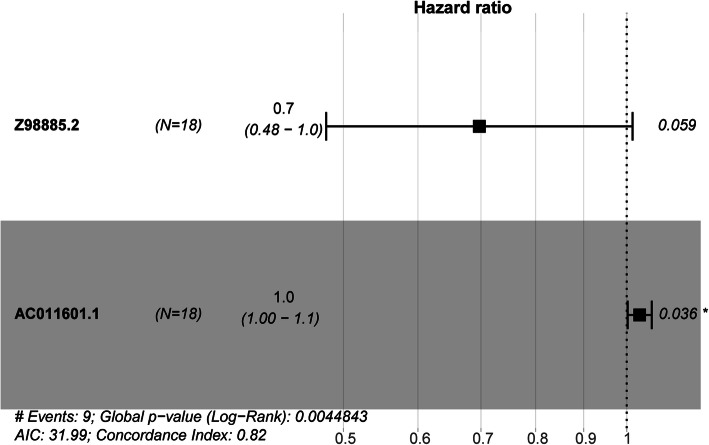
Identities of IncRNAs in the prognostic signature and their univariable cox association with prognosis

Univariate Cox hazards regression analysis was performed on the basis of differentially expressed lncRNA expression profiling data, using the overall status and survival time as the dependent variables. Four lncRNAs were strongly associated with recurrence (*P* < 0.05, Table S2). To select the most effective diagnostic lncRNAs, we performed multivariate Cox regression analysis (Fig. 3) and constructed a 2-lncRNA model to estimate the survival risk. The risk score (Table S3) of the combination, comprising Z98885.2 and AC011601.1, was determined as follows:

$$ RS=\left(-0.36\times {ev}_{Z9885.2}\right)+\left(0.032\times {ev}_{ac011601.1}\right) $$

where RS is the risk score, and ev is the expression value.

### Determining the survival power and adjuvant chemotherapy of the lncRNA gene signature in the dataset

**Fig. 4:**
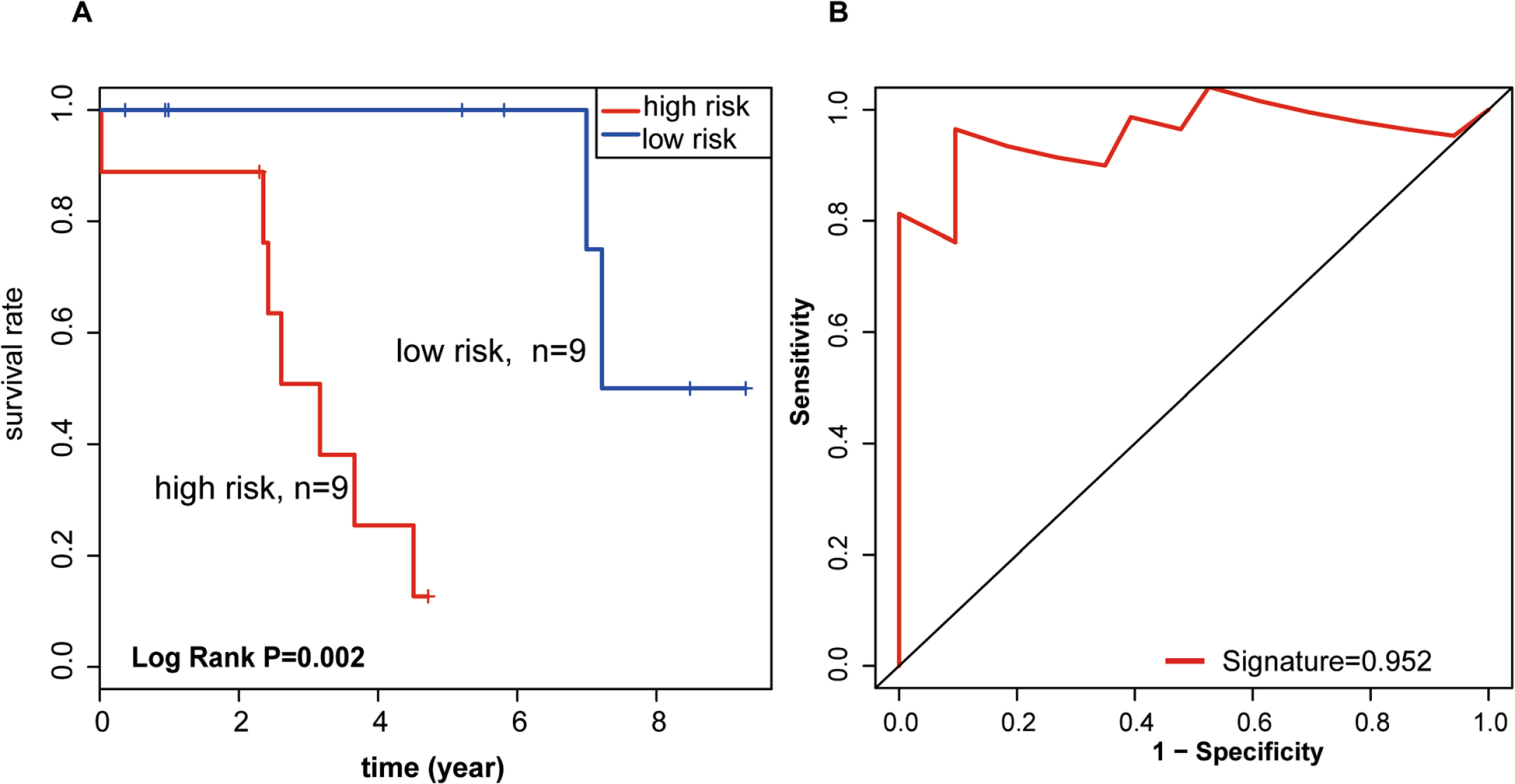
The IncRNA signature predicts the overall survival of patients with OC. **a**. Kaplan-Meier survival curves classified patients into high- and low-risk groups on the base of their lncRNA signatures in the sample datasets. *P*-values were determined through the log-rank test. **b**. Results of receiver operating characteristic (ROC) analysis

LncRNA markers were selected, and risk scores were allocated for each OV patient. OV patients were segregated into two group according to their risk score: low-risk (*n* = 9) and high-risk (*n* = 9) group. KM survival model analysis revealed that overall survival (OS) was considerably higher in the low-risk group than in the high-risk group (median survival: 7.2 years vs. 2.3 years (Fig. 4a, left). The 3-year OS of high-risk patients was almost 38% higher than that of the low-risk patients, approaching 100%.

**Fig. 5:**
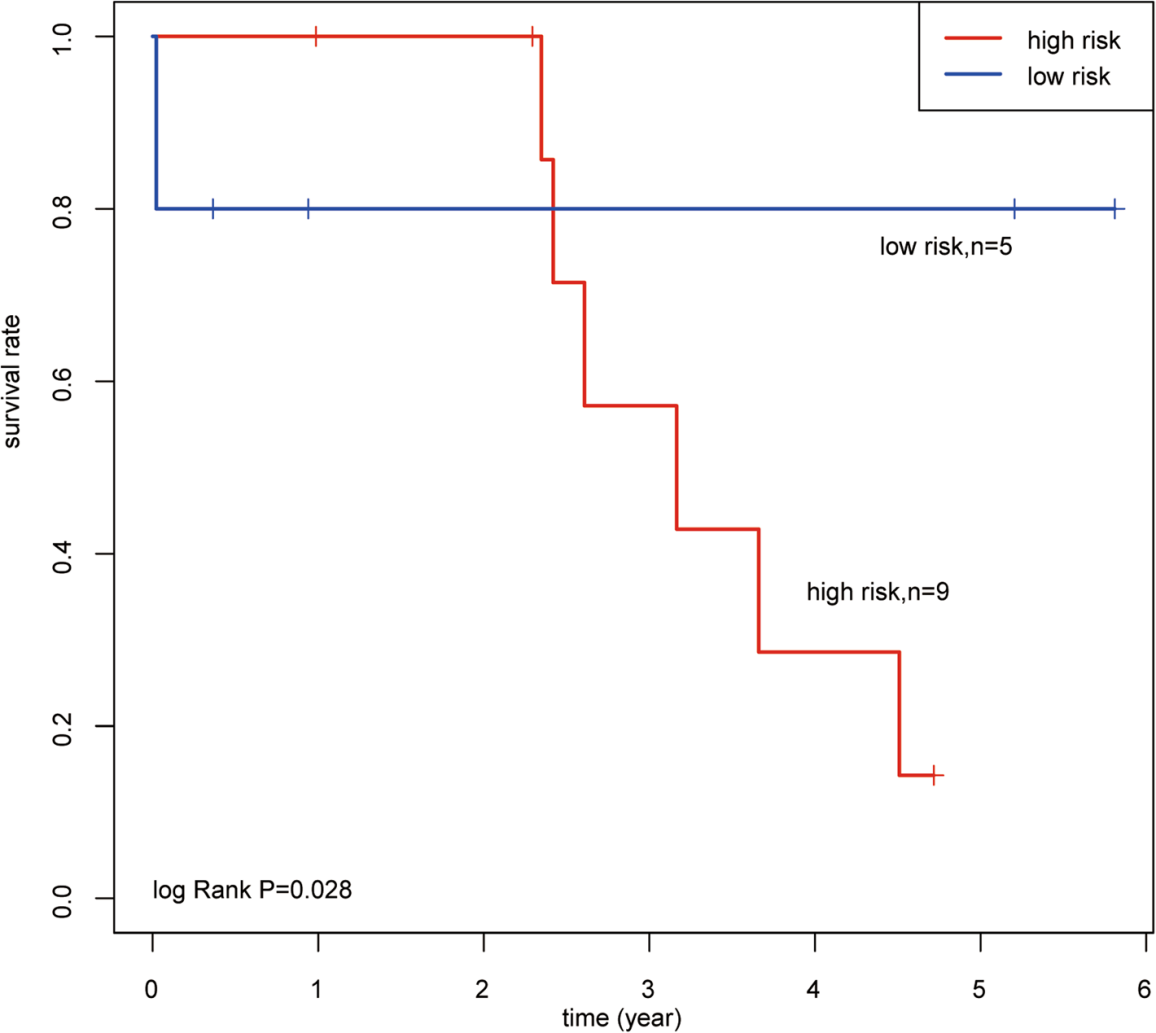
The IncRNA signature predicts the overall survival of chemotherapy treatment

To further understand whether the risk signature could be used to promote or reduce the efficacy of chemotherapy, KM survival model analysis was conducted between the low-risk (*n* = 5) and high-risk (*n* = 9) group (Fig. 5). The results showed that the high-risk group had significantly shorter OS with adjuvant chemotherapy compared to the low-groups. The overall one-, three- and five- year survival rates were 100, 40 and 15% respectively in the high-risk group; however, the low-risk groups had the same survival rate, of 80%.

ROC analysis was used to confirm the prognostic potential of lncRNA markers. A greater area under the ROC curve denotes a greater survival of patients harboring *BRCA1*/*2* mutations. The dataset supported the premise that the predictive value of the 2-lncRNA signature was high (AUC Signature = 0.952, Fig. 4b). These results suggest that the signature is a novel, highly accurate biomarker for survival.

### Functional enrichment analysis

**Fig. 6:**
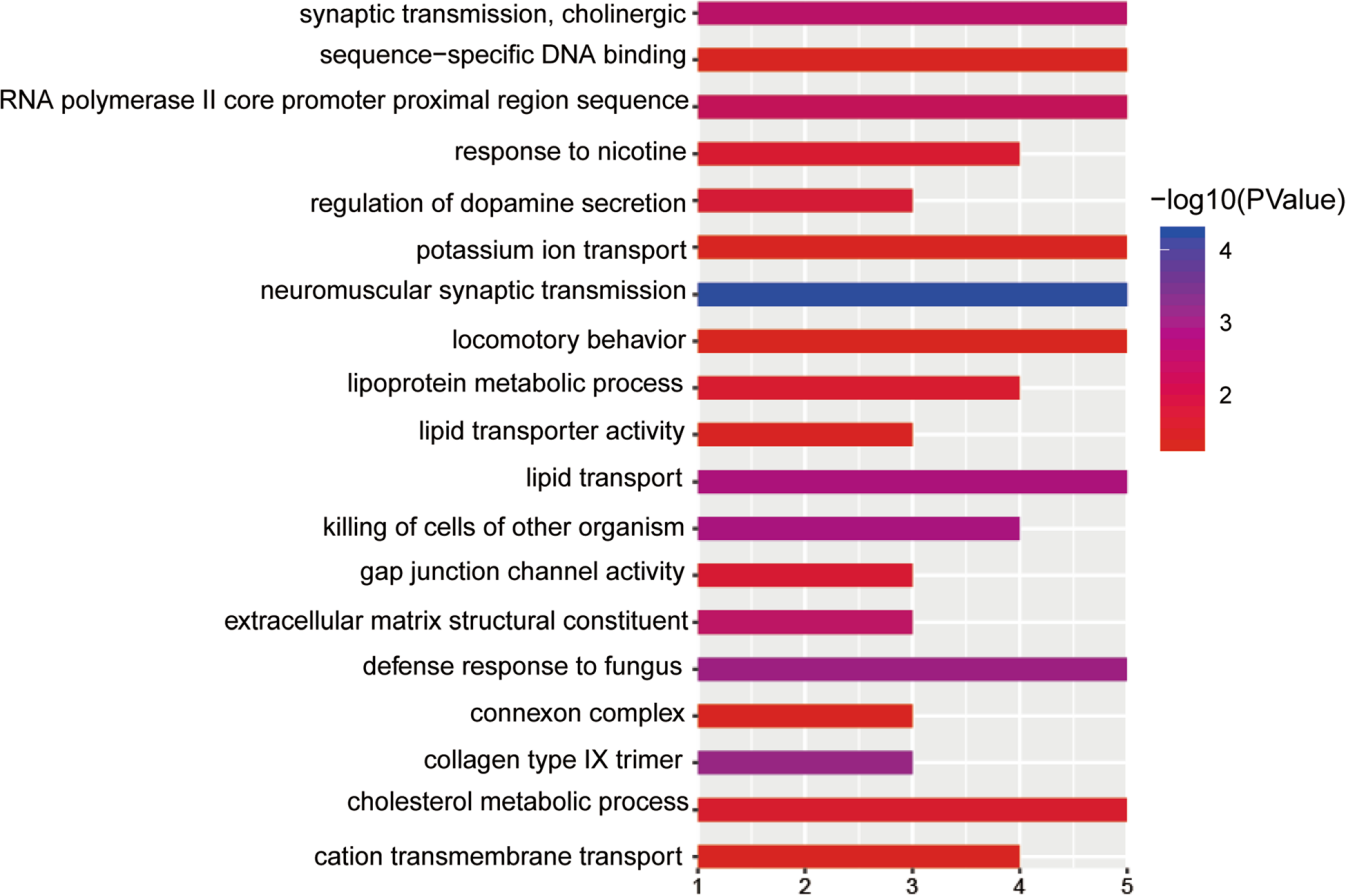
KEGG and GO analyses. KEGG and GO analyses were performed to investigate the potential involvement of different mRNAs in biological processes occurring in patients harboring *BRAC1* and *BRAC2* mutations

KEGG and GO analyses were used to investigate the potential involvement of different mRNAs in biological processes associated with patients with *BRCA1*/*2* mutations (Fig. 6, Table S4). The mRNAs were associated with biological processes including DNA binding, cholinergic neurotransmission, and lipid transport. Additionally, mRNAs that participate in MAPK/RAS and PI3K-Akt signaling pathways, which are critical for tumor development were identified.

### Nomogram development

**Fig. 7:**
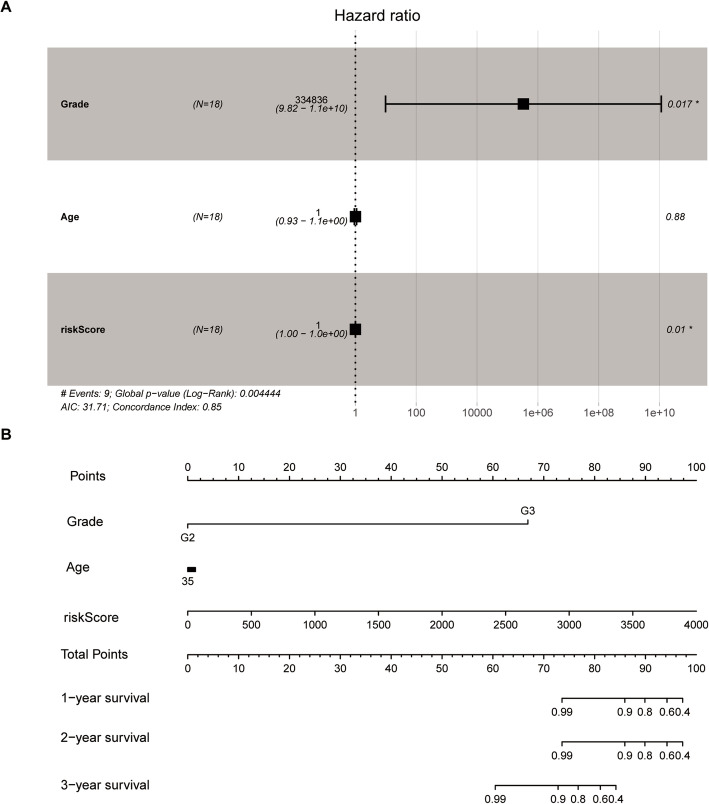
Multivariable Cox regression analysis and Nomogram to predict 3-year OS for OV patients. Multivariable Cox regression analysis was performed to assess the independence of the signature in survival prediction, and *P* value < 0.05 was considered significant. The nomogram was plotted using the rms package in R, including information such as age and stage in the nomogram, as they are usually included in most prognostic models of *BRAC1* and *BRAC2* muted groups

The aforementioned independence signatures, including tumor stage and age, each represented a point. Each point in the nomogram graph is indicated on the top scale (Fig. 7). The corresponding of one-, two-, and three-year survival rates were determined in accordance with the scale provided, and the predicted risk values for one-, two-, and three-year survival rates were predicted. The total score was determined by adding these values.

The respective point was determined to match the one-, two- and three- survival rates on the basis of the scale provided, predicting patients’ one-, two-, and three-year survival rates according to the risk prediction value, finally adding up to a total point. The C-index used in the nomogram was 0.952, and was used to predict the survival rate from the nomogram of OS patients.

## Discussion

OV has high mortality rates because the clinical symptoms of early OV are hard to detect, and in most cases, cancer has already advanced to late stages when diagnosed. Therefore, there is an urgent need to develop new targets for the treatment of OV [21, 22].

In this study, we used several different statistical tests to assess the risk signature of two lncRNAs and found that this risk signature was an independent factor capable of predicting *BRCA1*/*2* mutations in OV patients. A multivariate Cox regression model was applied to evaluate the independence of the signature, and to predict the prognostic potential of OV patients with mutations in *BRCA1*/*2*. Age and tumor grade were considered to be covariables in accordance with the risk scores of OV patients and were found to be independently associated with recurrence. Thus, we selected two lncRNAs, Z98885.2 and AC011601.1 as a risk signature.

An increasing number of studies have suggested that lncRNAs play important roles in the pathophysiology of OV amongst several other diseases. lncRNAs participate in a range of biological events and are known to regulate tumorigenic processes. To accurately predict the clinical outcomes or chemotherapy resistance of OV patients and improve their long-term survival, the development of novel molecular biomarkers for early OV detection is a high priority [23].

Xu Meng et al [24] identified a progressive transcription signature to predict the prognostic potential in OV, using protein-coding genes, lncRNAs, and miRNAs. Some lncRNAs, such as GAS5, rp11-190d6.2, and nbat-1 were downregulated in OV cells, and were significantly associated with histological grading, FIGO staging, and lymph node metastasis. Because 5–10% of OV is hereditary, the above observations are based on germline mutation, with only a few studies having evaluated mutant somatic genes [25].

Collectively, our results suggest that the two lncRNAs, Z98885.2 and AC011601.1, may serve as biomarkers to predict the survival of patients with OV. To date, the functions of Z98885.2 and AC011601.1 are relatively unknown. A previous study [26] reported the use of a tiling-path chromosome for the identification of limited regions of genetic aberration in patients affected with Wilm’s tumor. Four cases presenting presented with partial deletion or gain on chromosome 22 and Z98885–AC000036 was located, using an array-GGH profile, on a telomeric gain on chromosome 22. However, the study provided minimal information about the two lncRNAs described above. In contract, in this study, we provided comprehensive insights into the function of these lncRNAs.

*BRCA1*/*2* are major players in the machinery that repairs DNA double-strand breaks (DSBs) via homologous recombination (HR). Loss of *BRCA1*/*2* renders the cells HR-deficient, thus requiring the use of alternative, error-prone, repair pathways to fix DSB. The use of these alternative pathways can lead to chromosome deletions and translocations, and subsequent cell death. Women with *BRCA1* gene germline mutations have a 39% higher lifetime risk of OV, and those with *BRCA2* mutations have an 11% higher lifetime risk of OV [27]. Currently, PARPi, a potent drug against cancer caused by *BRCA* mutations leading to *BRCA* pathway defects, has attracted the attention of the pharmaceutical industry. PARPi leads to unmodified single-chain rupture (SSB) by inhibiting PARP activity and inducing DSB, while *BRCA* cascaded cells are unable to repair DSB through HR, resulting in cell death. PARPi also increases cell death by phosphorylating DNA-dependent proteases in non-homologous terminal junction pathways. PARPi has limited off-target side effects as it only targets tumor cells that simultaneously have *BRCA1/2* mutations, causing cell death [28]. Cells carrying *BRCA* mutations are up to 1000 times more sensitive to PARPi than are wild-type cells [29]. Olaparib was identified in stage I and II clinical trials as a single-agent to treat OV associated with *BRCA* mutations [30]. However, predicting the prognosis of OV patients with *BRCA1*/*2* mutations remains a challenge. Signatures that serve not only as biomarkers for the occurrence and development of OV, but also as therapeutic agents are urgently needed [31].

To confirm that signature can serve as a prognostic biomarker in OV patients, we calculated the risk score of the selected signature of OV for each patient. The median risk score separated the low-risk and high-risk group. The high-risk group had significantly higher disease progression rates than the low-risk group. Using data from the TCGA databases, the survival rate of the high-risk group was found to decline rapidly after 2 years of OA chemotherapy, while, an 80% survival rate for 5 years was observed in the low-risk groups. These results indicated that chemotherapy resistance may develop in the high-risk groups. The low-risk group was sensitive to platinum based chemotherapy treatment. Hence, the prognostic potential of lncRNA for OV patients harboring *BRCA1*/*2* mutations was considered to be an independent signature, distinct from miscellaneous clinical factors.

To further evaluate of the differences between *BRCA1/ 2* mutations, we identified 117 and differentially expressed mRNAs and 325 differentially expressed lncRNAs from 19 *BRCA1/2* mutations. GO and KEGG analyses indicated that these genes are involved in the MAPK/RAS and PI3K-Akt signaling pathways. MAPK and PI3K-Akt are responsible for sustained proliferative signaling, while RAS participates in the inflammatory response. Each of these pathways is closely associated with tumorigenesis and tumor progression.

The nomogram model is considered to be an evidence-based, accurate method for the assessment of treatment and prognosis, and has been widely used in studies on a variety of malignant tumors [32, 33]. The progressive potential of the clinical model was assessed using the C-index by multivariate Cox regression analysis with matched OV patients [34]. A nomogram prediction model was successfully constructed on the base of independent risk factors determined through survival analyses. By incorporating independent risk factors into nomogram modeling to predict the survival rate, a C-index of 0.952 was achieved, indicating the excellent predictive ability of this method. The model can predict the survival rate of individual patients and is helpful for clinical treatment decision-making and design of clinical research programs.

Some limitations of this study must be acknowledged. First, we investigated only a fraction of the lncRNA expression dataset. Our dataset was not large enough to validate the independent lncRNA signature for survival prediction using a test group. Second, the prognostic lncRNAs defined here may be accompanied by other, yet unidentified, lncRNA candidates. Second, we only provided a limited mechanistic explanation of the roles played by the two lncRNAs in OV. Further experimental studies on the lncRNAs are needed to deepen our understanding of their functional mechanisms. Third, our clinical data on TCGA ovarian cancer did not include such clinical data as disease stage, surgical residual tissue or histological type, so these were not taken into account. However, we collected clinical data for ovarian cancer patients with a mutation in BRAC1/2, and verified whether the signature we developed could distinguish different tumor stages, post-operative residual disease and histologic types. Notwithstanding these limitations, the robust and consistent correlation observed in this study between two lncRNA biomarkers and overall survival indicates that this biomarker is a dominant independent signature for OV.

## Conclusion

In conclusion, this study shows that a signature consisting of two lncRNAs has potential clinical value for the early diagnosis and prognostic monitoring of ovarian cancer. Future studies evaluating the mechanisms involved in ovarian cancer will provide a theoretical basis for the development of successful targeted therapy.

## Supplementary information

**Additional file 1:** **Figure S1**. Volcano plot of mRNAs and IncRNAs. Differentially expressed mRNAs and lncRNAs, Fold changes (log2 absolute) ≥2, *P* < 0.05 and FDR < 0.05 indicated a statistically significant difference.

**Additional file 2:** **Table S1**. Differentially expressed mRNAs and lncRNAs.

**Additional file 3:** **Table S2**. Univariate Cox proportional hazards regression analysis (*P* < 0.05) of the differentially expressed lncRNAs profiling data in the dataset.

**Additional file 4:** **Table S3**. The signature risk score composed of 2 lncRNAs combinations in the dataset.

**Additional file 5:** **Table S4**. Functional enrichment analysis of different mRNAs.

## Data Availability

The dataset supporting the conclusions of this article is included within the article.
