# Supplementary figures and images for: A novel signature of two long non-coding RNAs in BRCA mutant ovarian cancer to predict prognosis and efficiency of chemotherapy
Source: J Ovarian Res. 2020 Sep 19;13:112. doi: 10.1186/s13048-020-00712-w (PMC7502206; doi:10.1186/s13048-020-00712-w)

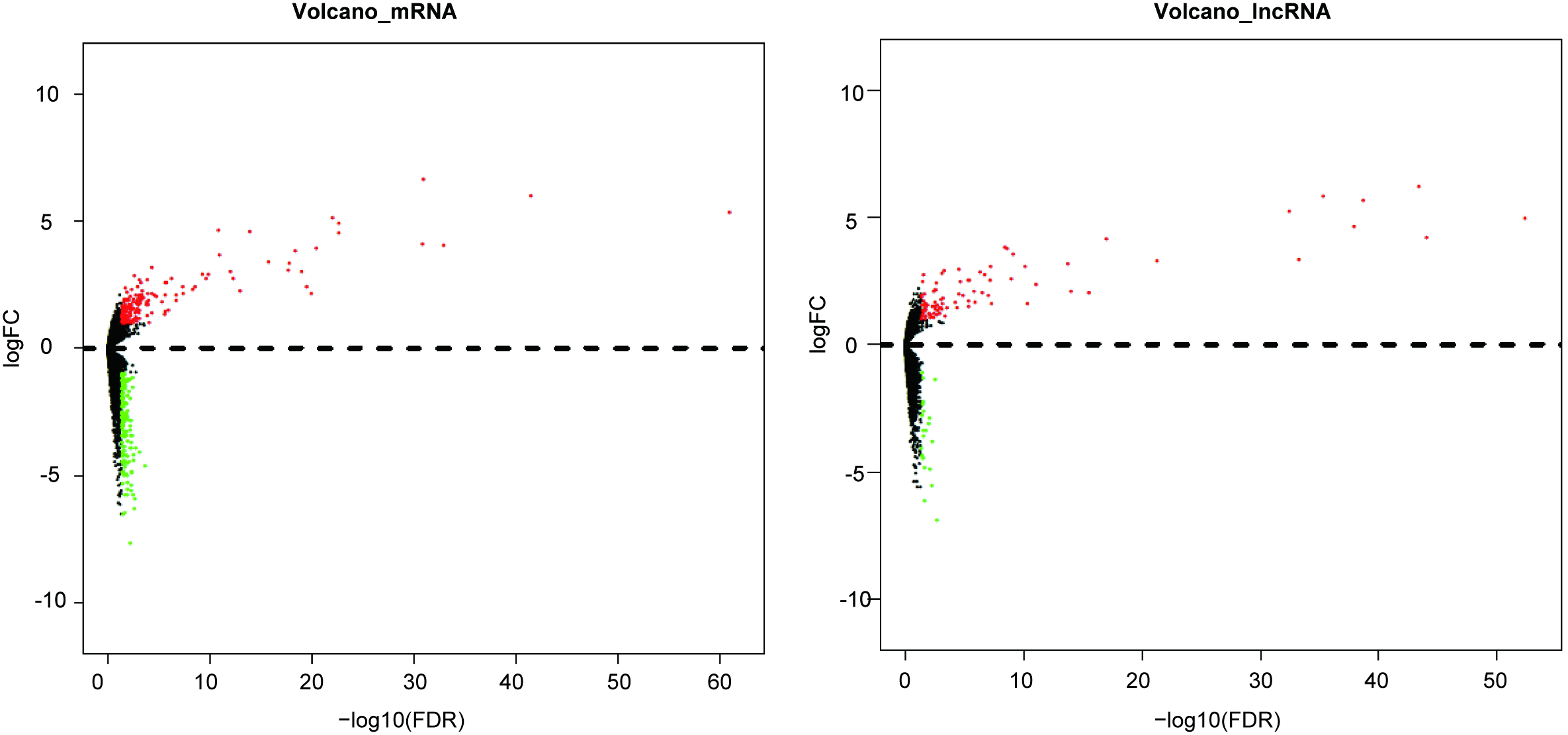

Supplement: Supplementary file 1 — Additional file 1: Figure S1. Volcano plot of mRNAs and IncRNAs. Differentially expressed mRNAs and lncRNAs, Fold changes (log2 absolute) ≥2, P < 0.05 and FDR < 0.05 indicated a statistically significant difference. [file 13048_2020_712_MOESM1_ESM.tif]
